# Cardiac Migration of Endogenous Mesenchymal Stromal Cells in Patients with Inflammatory Cardiomyopathy

**DOI:** 10.1155/2015/308185

**Published:** 2015-02-28

**Authors:** Caroline Schmidt-Lucke, Felicitas Escher, Sophie Van Linthout, Uwe Kühl, Kapka Miteva, Jochen Ringe, Thomas Zobel, Heinz-Peter Schultheiss, Carsten Tschöpe

**Affiliations:** ^1^Department of Cardiology, Charité–University Medicine Berlin, Campus Benjamin Franklin, 12200 Berlin, Germany; ^2^Berlin-Brandenburg Center of Regenerative Therapies (BCRT), 13353 Berlin, Germany; ^3^Medico-Academic Consultings (MEDIACC), 14193 Berlin, Germany; ^4^Department of Cardiology, Charité–University Medicine Berlin, Campus Rudolf Virchow, 13353 Berlin, Germany; ^5^German Center for Cardiovascular Research (DZHK), 10117 Berlin, Germany; ^6^Institute of Cardiac Diagnostics and Therapy (IKDT), 12203 Berlin, Germany

## Abstract

*Introduction*. Mesenchymal stromal cells (MSC) have immunomodulatory features. The aim of this study was to investigate the migration and homing potential of endogenous circulating MSC in virus negative inflammatory cardiomyopathy (CMi). *Methods*. In 29 patients with (*n* = 23) or without (*n* = 6) CMi undergoing endomyocardial biopsies (EMB), transcardiac gradients (TCGs) of circulating MSC were measured by flow cytometry from blood simultaneously sampled from aorta and coronary sinus. The presence of MSC in EMB, cardiac inflammation, and SDF-1*α* mRNA expression were detected via immunohistochemistry and real-time PCR. *Results*. MSC defined as CD45^−^CD34^−^CD11b^−^CD73^+^CD90^+^ cells accounted for 0.010 [0.0025–0.048]%/peripheral mononuclear cell (PMNC) and as CD45^−^CD34^−^CD11b^−^CD73^+^CD105^+^ cells for 0.019 [0.0026–0.067]%/PMNC, both with similar counts in patients with or without cardiac inflammation. There was a 29.9% (*P* < 0.01) transcardiac reduction of circulating MSC in patients with CMi, correlating with the extent of cardiac inflammation (*P* < 0.05, multivariate analysis). A strong correlation was found between the TCG of circulating MSC and numbers of MSC (CD45^−^CD34^−^CD90^+^CD105^+^) in EMB (*r* = −0.73, *P* < 0.005). SDF-1*α* was the strongest predictor for increased MSC in EMB (*P* < 0.005, multivariate analysis). *Conclusions*. Endogenous MSC continuously migrate to the heart in patients with CMi triggered by cardiac inflammation.

## 1. Introduction

Despite advances in medical procedures, the prognosis of inflammatory cardiomyopathy (CMi), a cause for dilated cardiomyopathy, is poor [1]. The character of inflammatory response is the pivotal parameter for the prognosis of patients with CMi [2]. The delicate balance between protective and deleterious immune mechanisms is a decisive factor in the evolution of myocardial damage [3], of which the complex mechanisms involved are not yet completely understood. Beyond others, the innate immune system [4], cardiac cytokines, autoantibodies, and probably also mesenchymal stromal cells (MSC) [5] are involved in this process. MSC are of special interest, since they produce growth factors and cytokines [6] mediating endogenous activation of resident cardiac stem cells [7] in a paracrine manner, and have profound immunomodulatory features [8]. MSC mediate their complex immunomodulatory effects by interacting with cells from both the innate and adaptive immune system resulting in skewing the immune response towards an anti-inflammatory/tolerant phenotype [9]. They suppress T-cell proliferation [10], modulate dendritic cell function into a regulatory phenotype, and alter their cytokine secretion profile towards an upregulation of anti-inflammatory cytokines such as interleukin- (IL-) 10 and downregulation of inflammatory cytokines [11]. MSC counteract tissue injury via their capacity to promote endogenous tissue regeneration and via immunomodulation after selective migration to the site of injury, a process in which stromal derived factor- (SDF-) 1*α* is essential [12]. This feature supports the intravenous (i.v.) administration of MSC as application route in CMi, whose efficacy has been demonstrated in experimental studies of acute myocarditis [8, 13]. At present, no clinical studies have yet been performed treating patients with CMi by i.v. administration of MSC. Before starting clinical i.v. application of MSC in patients with CMi, clarification in the potential of MSC to migrate and home to the heart in patients with CMi is warranted. Homing is a complicated process orchestrated by distinct chemokines. First, regenerating cells are recruited to the site of damage; thereafter they adhere to the vessel wall or migrate into the tissue. Thereafter, SDF-1*α* helps to retain cells in the tissue. Therefore, the aim of this study was to investigate the migration and homing potential of endogenous circulating MSC in patients with virus negative CMi.

## 2. Methods

### 2.1. Patients and Control Subjects

Patients with clinical indication for an EMB were consecutively recruited between November 2008 and June 2009 in our own centre. Inclusion criteria were age between 18 and 75 years. Indications for EMB were reduced ejection fraction in the presence of a normal coronary angiogram (*n* = 16), symptoms of heart failure despite normal left ventricular ejection fraction (LVEF) (*n* = 7), LV hypertrophy (*n* = 2), or cardiac arrhythmias (*n* = 4). We excluded patients with proof of virus genomes in EMB of Human Herpes virus 6, Epstein Barr virus, Enterovirus, Adenovirus, and Parvovirus B19, for the latter with genome equivalence >500/*μ*g. In addition, exclusion criteria were coronary heart disease, antiviral, immunomodulatory, or immunosuppressive therapy within the last 6 months, acute myocarditis, clinical or biochemical evidence of the presence of concomitant chronic inflammatory disease, chronic renal insufficiency (creatinine ≥ 1.4 mmol/L), inability to understand the consent form, participation in or consent to participate in another study, or malignant disease. All study participants gave their written informed consent, and the study was approved by the ethics committee of the Charité, Berlin.

Physical examinations, clinical assessments including echocardiography and ECG, as well as laboratory controls were analysed prospectively. The assessment of clinical complaints and heart failure symptoms according to the NYHA classification and the completion of a questionnaire were carried out at the initial visit.

LVEF was evaluated by LV angiography at the point of EMB [14]. All patients underwent M-mode and 2D echocardiography to evaluate LV end diastolic diameter (LVEDD). Echocardiographic measurements were analysed independently in a blinded fashion by two experienced operators.

### 2.2. Analysis of Surface Markers for Circulating Mesenchymal Stromal Cells

Peripheral blood mononuclear cells (PBMC) were isolated using a Ficoll gradient (Histopaque 1077, Sigma). PBMC were analysed by flow cytometry. First, a regional gate was defined to exclude debris and platelets defined by forward/sideward scatter. In order to detect MSC, CD45^−^CD34^−^CD11b^−^ events identified with directly conjugated monoclonal antibodies against human CD45, CD34 (both FITC, BD), and CD11b (AF488, BD) were used. A total of at least 100,000 events in R1 + R2 were counted. Cells were identified in a forward/sideward scatter (R1). They were negative for this marker combination (R1 + R2) and further analysed for the presence of CD105 or CD90 (both APC, BD) and CD73 (PE, BD). However, due to the rare counts of cells fulfilling these criteria in a number of patients, further statistical analysis was hindered. Thus, due to the very close correlation of CD45^−^CD34^−^CD11b^−^ with CD45^−^CD34^−^CD11b^−^CD73^+^CD90^+^ (*r* = 0.67, *P* < 0.0001) and with CD45^−^CD34^−^CD11b^−^CD73^+^CD105^+^CD90^+^ (*r* = 0.72, *P* < 0.0001), we chose to use CD45^−^CD34^−^CD11b^−^ cells for further analysis. Isotype-identical antibodies served as controls (Becton Dickinson). Measurements were carried out in duplicate and the mean value was used for further calculations. All antibodies were tested with cultivated MSC on passage 6 and with cultivated fibroblasts for their sensitivity and specificity.

### 2.3. Transcardiac Gradients of Circulating Mesenchymal Stromal Cells in Inflammatory Cardiomyopathy

As described previously [15], all patients underwent sampling from the femoral vein and great cardiac vein for measurement of MSC and cytokine levels before an EMB specimen was taken. In patients scheduled for coronary angiography, additional blood samples were obtained from the aortic root before injection of the contrast agent. The transcardiac gradients (TCGs) were expressed in percent changes from values obtained from either the aortic root or, if not obtainable there, the femoral vein. A “negative” transcardiac gradient thus means that the number of cells is smaller in the coronary sinus compared to the aortic root and suggests that cells are retained in the heart. There were no differences in TCGs between the 2 methods. In patients, in whom both aortic root and femoral vein were sampled, comparable values were obtained from the latter sampling sites.

### 2.4. Histological and Immunohistological Evaluation of Inflammation in Endomyocardial Biopsies

EMB were obtained from the right ventricular septum within two days after angiographic evaluation of LVEF using standard techniques [16]. Two biopsies were used for the histological evaluation (paraffin embedded) according to the Dallas criteria [17] and the immunohistological analysis of inflammation (frozen sections), respectively [18]. The cut-off value of 9.0 lymphocyte function-associated antigen-1 (LFA-1, CD11a) positive cells/mm² and 5.5 area fraction (AF)% human leucocyte antigen (HLA) class I and/or 1.2 AF% CD54/ICAM-1 were used to differentiate between samples with or without cardiac inflammation. DNA and RNA were extracted simultaneously from biopsy specimens, and the amplification of viral genomes was conducted by nested PCR as described [16]. LFA-1, HLA class I and CD54/ICAM-1 were detected immunohistochemically as described previously.

### 2.5. NT-proBNP and SDF-1*α* Analysis

Circulating levels of N-terminal pro-brain natriuretic peptide (NT-proBNP) and SDF-1*α* levels in pre- and postcoronary blood samples were quantified with respective ELISA kits (R&D, Wiesbaden) according to the manufacturers' instructions.

### 2.6. Real-Time Polymerase Chain Reaction Quantification

RNA from human biopsies was prepared and reverse transcribed using High Capacity cDNA Reverse Transcription Kit (Applied Biosystems, Foster City, USA). Quantitative real-time polymerase chain reaction (PCR) was used to quantify human SDF-1*α* (ABI PRISM 7900 HT Sequence Detection System software version 2.2.2., Perkin Elmer) (Eppendorf Mastercycler epgradient realplex, Hamburg, Germany) expression. Expression levels were normalized to human ribosomal L32 in all experiments. The sequences of the primer sets used in this study were as follows: human SDF-1*α* FOR: 5′-ATGCCCATGCCGATTCTTC-3′ and REV: 5′-GGAGTGTTGAGAATTTTGAGATGCT-3′, and human ribosomal L32: FOR: 5′-AGGAGAGACACCGTCTGAACAAG-3′ and REV: 5′-GAACCAGGATGGTCGCTTTC-3′.

### 2.7. Immunofluorescence Analysis of Mesenchymal Stromal Cells in Endomyocardial Biopsies

MSC were determined in LV sections with transversely sectioned cardiomyocytes immunostained with CD105 and CD90 in the absence of the markers CD45 and CD34. The ratio of MSC to total nuclei of cardiomyocytes was calculated. High power fields (×400, 200 *μ*m × 200 *μ*m, 8 fields per section) with transversely sectioned cardiomyocytes were recorded to calculate MSC to cardiomyocyte ratio. Nuclei were stained with 4′,6-diamino-2-phenylindole (DAPI, dilution, 1 : 200; Sigma). Ki-67 in nuclei was evaluated with the use of anti-Ki-67 antibodies (Diagnostic Biosystems, Pleasanton, USA). Myocytes were recognized by means of mouse monoclonal antibodies against sarcomeric *α*-actin (dilution 1 : 50, Sigma). Finally, sections were mounted and examined under a fluorescence microscope (Leica). All available fields (40 fields) were measured.

### 2.8. Mesenchymal Stromal Cell Isolation

**Figure 1:**
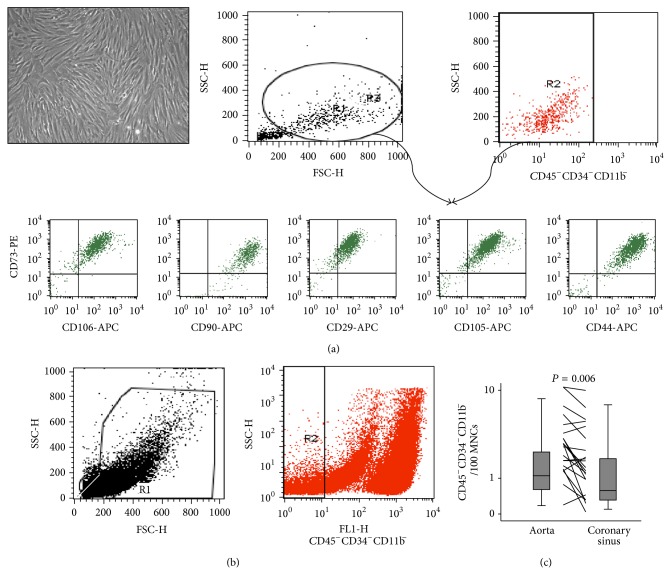
Flow cytometry analysis strategy and quantification of transcardiac gradients of circulating MSC in CMi. (a) Cultivated MSC (bar indicates 200 *μ*m) isolated from bone marrow were stained with directly FITC-conjugated monoclonal antibodies against human CD45 and CD34, and anti-CD11b directly conjugated to AF488 (all from BD). After setting a morphological gate defined by forward/side scatter (FSC and SSC, R1), CD45^−^CD34^−^CD11b^−^ cells (R2) defined as belonging to both R1 and R2 (first row) were stained with CD73-PE, CD106-APC, CD90-APC, CD29-APC, CD105-APC, or CD44-APC (second row). (b) Measurement of circulating MSC in patients with CMi. MNCs were isolated from peripheral blood using a Ficoll gradient. Circulating MSC were analysed by flow cytometry. First, a regional gate was defined to exclude debris and platelets defined by forward/side scatter (FSC and SSC, R1). The events in R1 are then displayed on a CD45CD34CD11b versus SSC dot plot and a second gate (R2) is produced to include the cluster of CD45^−^CD34^−^CD11b^−^ cells. The right margin was fixed for all patients. A total of at least 100,000 events in R1 and R2 were counted. (c) Quantification of the number of circulating MSC sampled from the aortic root and simultaneously from the coronary sinus in patients with cardiac inflammation (box plots are given as median and IQR; whiskers represent 95% CI). The lines between the boxes represent numbers of CD45^−^CD34^−^CD11b^−^/100 MNC sampled from either the aorta or coronary sinus of the same individual.

Human adult MSC were isolated from iliac crest bone marrow aspirates of healthy donors after their written approval according to Binger et al. [19]. MSC were cultured in Dulbecco's Modified Eagle's Medium (DMEM; Biochrom) supplemented with 10% fetal bovine serum (FBS), 1% penicillin/streptomycin, 1% glutamine, 2% HEPES, and 2 ng/mL of basic fibroblast growth factor (Tebu-bio, Offenbach, Germany) and plated at a density of 5 × 10^3^ cells/cm^2^ before use in the respective experiments. Cultivated MSC were triple negative for the markers CD45, CD34, and CD11b but stained positively for the markers CD73, CD29, CD105, CD106, CD90, and CD44, thereby confirming sensitivity and specificity of our antibody sets (Figure 1(a)).

### 2.9. Migration Assay

HL-1 cells were plated in a 6-well plate at a density of 300,000 cells/well in Claycomb medium (Sigma) supplemented with 10% FBS, 1% penicillin/streptomycin, 100 *μ*M norepinephrine (Sigma), and 2 mM L-glutamine (Biochrom AG, Berlin, Germany). After twenty-four hours, cells were washed and cultured for an additional 24 h in serum starvation medium (DMEM 11966 medium (Invitrogen, Carlsbad) supplemented with 0.1% FBS, 5 mM glucose, and 1% penicillin/streptomycin) in the presence or absence of 10 ng/mL of recombinant TNF-*α* (R&D Systems). Then, the supernatant (conditioned medium) was collected and stored at −20°C until further use.

Conditioned medium of unstimulated or TNF-*α*-stimulated HL- 1 cells, in the presence or absence of 5 *μ*g/mL of anti-SDF-*α* (R&D Systems) and corresponding mouse isotype control (Santa Cruz Biotechn Inc., Cambridge, USA) or 2.5 *μ*g/mL of anti-MCP-1 (R&D Systems) and corresponding rat isotype control (Santa Cruz Biotechn Inc.) was added to the bottom chamber of the chemotaxis chamber (ChemoTX, 8 *μ*M pore, Neuroprobe, Gaithersburg, MD, USA). A total of 3 × 10^4^ MSC, resuspended in DMEM (Sigma), containing 0.01% FBS, 2 mM L-glutamine, 20 mM HEPES (Invitrogen), and 1% penicillin/streptomycin, were plated in the upper chemotaxis chamber. After 24 hours of incubation at 37°C, the lower side of the filter was washed with PBS and fixed in 50% acetone/50% methanol. Next, cells were stained with crystal violet (Sigma) and quantified by spectophotometric measurement at 560 nm. Data are depicted as the migration of MSC versus conditioned medium of unstimulated HL-1 cells (basal) set as 1 and represented as mean ± SEM of *n* = 4 wells/condition.

In the case of migration assays of MSC towards serum of patients, 1% of patient serum was added to the bottom chamber of the chemotaxis chamber (ChemoTX, 8 *μ*M pore, Neuroprobe, Gaithersburg, MD, USA) in the presence or absence of anti-SDF-*α* and corresponding mouse isotype control. A total of 3 × 10^4^ MSC, resuspended in DMEM (Sigma), containing 0.01% FBS, 2 mM L-glutamine, 20 mM HEPES (Invitrogen), and 1% penicillin/streptomycin, was plated in the upper chemotaxis chamber. After 24 hours of incubation at 37°C, the lower side of the filter was washed with PBS and fixed in 50% acetone/50% methanol. A MSC standard was plated out on a separate membrane and after 2 hours, the membrane was fixed in 50% acetone/50% methanol. Next, cells were stained with crystal violet (Sigma). The optical density of the wells on the filter was analyzed via Image J and the corresponding cell number per well calculated. Data are depicted as migrated cells.

### 2.10. Statistical Analysis

Continuous variables were tested for normal distribution with the Kolmogorov-Smirnov test. Comparisons between the two groups were analysed by *t*-test (two-sided) for normally distributed variables. Data are expressed as mean ± SD, unless otherwise stated. Nonnormally distributed continuous variables (MSC, hs-CRP, LFA-1, NT-proBNP) were compared by the Mann-Whitney *U* test and are presented as median and interquartile range [IQR]. Comparison of categorical variables was generated by the Pearson *χ* ^2^ test. Multivariate linear regression analysis and nonparametric bivariate correlation (Spearman rank correlation coefficient) were used to correlate counts of circulating MSC or MSC in EMB with cardiac inflammation, NTproBNP, SDF-1*α*, MCP-1, and LVEF. To identify independent determinants of MSC counts, a multivariate linear regression analysis for the abovementioned parameters was performed.

Statistical significance was assumed, if a null hypothesis could be rejected at *P* ≤ 0.05. All statistical analysis was performed with SPSS 21 (SPSS Inc.).

## 3. Results

### 3.1. Patient Characteristics

The baseline characteristics of the 29 subjects are summarised in Table 1. Twenty-three (79%) patients showed an increased cardiac inflammation response in EMB. Patients with cardiac inflammation had a similar LVEF and a slightly increased LVEDD compared to patients without inflammation. Except for these, no differences regarding concomitant medication, age, gender, or duration of symptoms were apparent.

### 3.2. Circulating Mesenchymal Stromal Cells in Humans

Circulating MSC defined by the absence for the markers CD45, CD34, and CD11b and by staining positive for CD73, CD105, and CD90 counted for 0.010 [0.0025–0.048]%/PMNC with similar counts in patients with or without cardiac inflammation. When considering only the negative marker combination (Figure 1(b)), MSC comprised 0.62 [0.38–1.15]%/PBMCs, again with similar counts in patients with or without cardiac inflammation (0.52 [0.29–1.0] versus 0.76 [0.38–1.34]%/PBMC). No correlation was seen between circulating MSC and cardiac inflammation, LVEF, leukocyte count, or hs-CRP, indicating that inflammation* per se* has no direct effect on circulating MSC counts.

### 3.3. Transcardiac Gradients of Circulating Mesenchymal Stromal Cells in Patients

**Figure 2:**
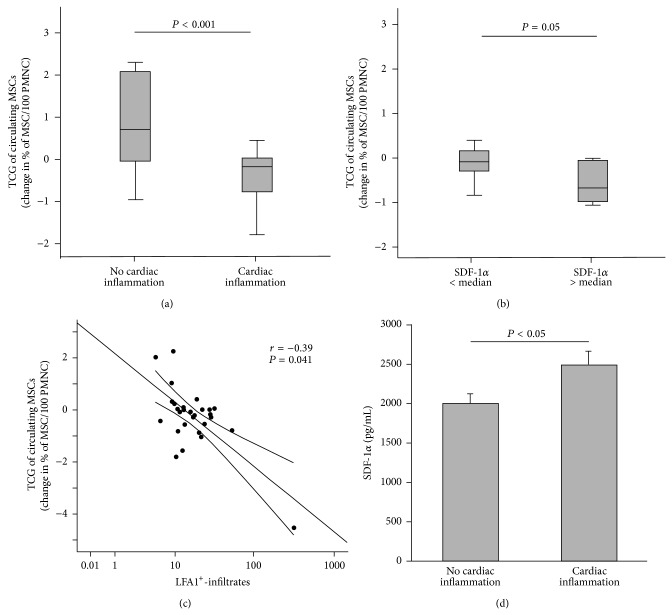
Transcardiac gradients of circulating MSC and Stromal derived factor-1*α* (SDF-1*α*) related to cardiac inflammation and expression of SDF-1 mRNA. (a) Quantitative analysis of transcardiac gradients of MSC in patients without (*n* = 6) and with (*n* = 23) cardiac inflammation, as defined by the number of LFA-positive cells, is given as median and IQR; whiskers represent 95% CI. (b) Transcardiac gradient of MSC after dichotomisation with respect to SDF-1*α* mRNA in their EMB (cut-off: median, box plots are given as median and IQR; whiskers represent 95% CI). (c) Correlation between transcardiac gradients of MSC and cardiac inflammation. (d) Postcoronary SDF-1*α* levels (pg/mL) in patients without and with cardiac inflammation; bar graphs represent the mean ± SEM.

Transcardiac gradients of circulating MSC were measured after simultaneous sampling of the aorta and the coronary sinus in all patients. As illustrated in Figure 2(a), patients with cardiac inflammation (*n* = 23) had a significant 29.9% reduction of MSC after transcardiac passage (*P* < 0.01, Figure 1(c)), in contrast (*P* < 0.001) to patients without cardiac inflammation (*n* = 6). Since SDF-1*α* is a potent chemoattractant for MSC [12], SDF-1*α* mRNA was quantified in EMB. There was a negative transcardiac gradient of MSC in patients with SDF-1*α* mRNA in their EMB > median (>2.7-fold compared to GAPDH; *P* < 0.05, Figure 2(b)). Cardiac inflammation and SDF-1*α* mRNA levels were inversely correlated with the transcardiac gradients of circulating MSC (cardiac inflammation: *r* = −0.39, *P* < 0.05, Figure 2(c)). Furthermore, there was a negative correlation between the transcardiac gradients of circulating MSC and NT-proBNP (*r* = −0.46, *P* < 0.05, data not shown), but not with LVEF. By multivariate analysis, including LVEF, NT-proBNP, cardiac inflammation (LFA-macrophages), and cardiac SDF-1*α* mRNA, cardiac inflammation remained the only significant independent predictor for declined numbers of circulating MSC after transcardiac passage (*P* < 0.001 for trend (ANOVA), *B* = −0.73, *P* < 0.005). Interestingly, systemic SDF-1*α* levels were significantly induced in patients with cardiac inflammation compared to patients without cardiac inflammation (Figure 2(d)) with no further increase after transcardiac passage in both, patients with or without cardiac inflammation. Furthermore, MSC migration was only tendentiously (*P* = 0.1) more pronounced versus postcoronary or venous serum of patients with cardiac inflammation compared to serum of patients without inflammation (data not shown). However, no SDF-1*α*-dependency could be detected, as shown in a blocking experiment (data not shown).

### 3.4. Mesenchymal Stromal Cells in Human Endomyocardial Biopsies

**Figure 3:**
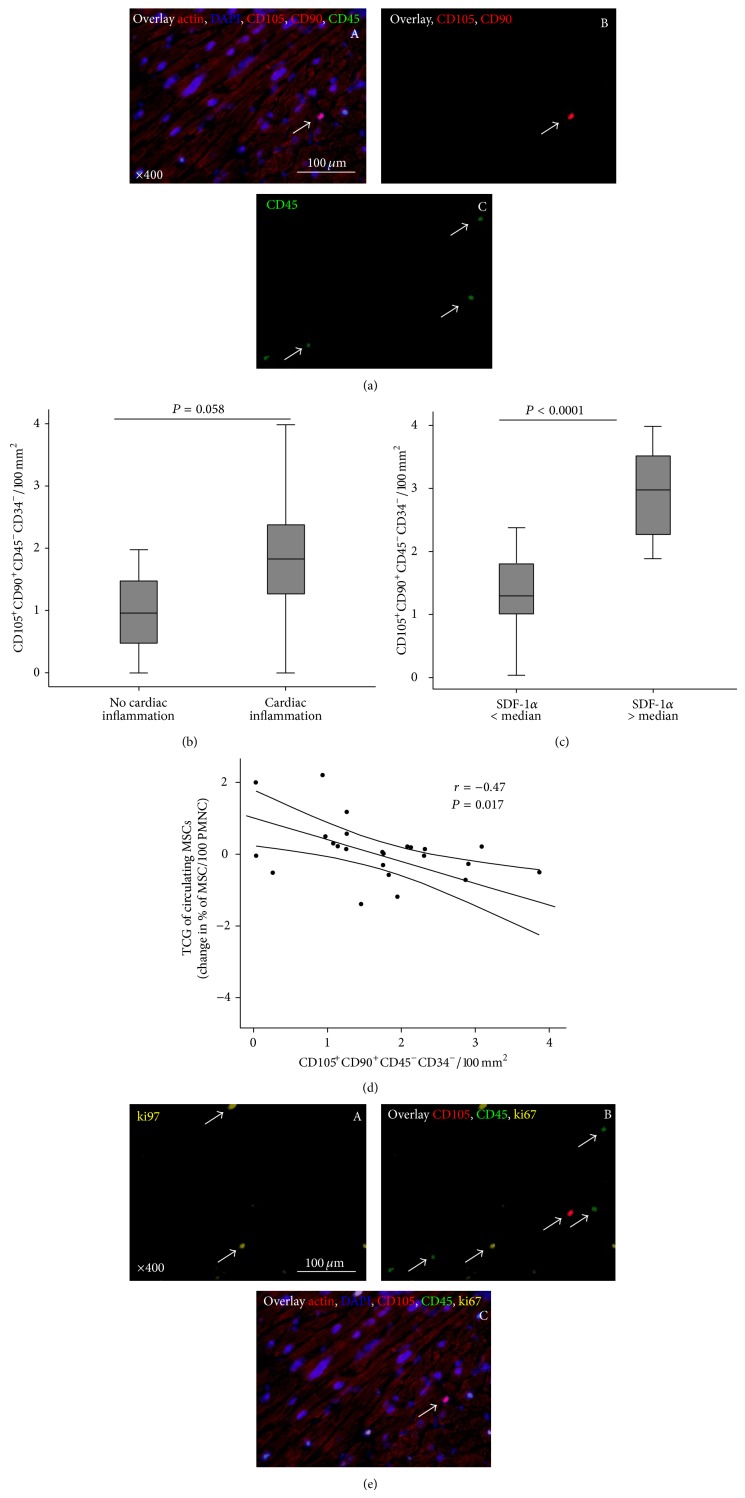
Immunostaining and quantification of cardiac MSC related to cardiac inflammation and expression of SDF-1*α* mRNA. (a) Representative image of immunofluorescence of MSC in EMB. Serial sections were stained with (A) an overlay of *α*-sarcomeric actin (red), nuclear counterstained (DAPI, blue), anti-CD105 and anti-CD90 (red, and B), and anti-CD45 (green, and C) as a lymphocytes marker (the arrow image (A) highlights the MSC). (b) Quantitative analysis of CD45^−^CD34^−^CD105^+^CD90^+^-MSC in EMB of patients without (*n* = 6) and with (*n* = 23) cardiac inflammation, as defined by the number of LFA-positive cells (box plots are given as median and IQR; whiskers represent 95% CI). (c) Quantitative analysis of CD45^−^CD34^−^CD105^+^CD90^+^-MSC in EMB after dichotomisation with respect to SDF-1*α* mRNA expression in their EMB (cut-off: median, box plots are given for median and IQR; whiskers represent 95% CI). (d) Correlation between the transcardiac gradient of circulating MSC and MSC in EMB. (e) Representative image of immunofluorescence of MSC and proliferation in EMB. The same serial sections as indicated above (3(a)) were stained with (A) anti-Ki67 (yellow) for proliferation, (B) overlay of CD105 (red), anti-CD45 (green), and Ki67 (yellow), and (C) overlay of *α*-sarcomeric actin (red), nuclei (DAPI, blue), CD105 (red), anti-CD45 (green), and Ki67 (yellow).

As shown in the representative image, MSC can be identified in the myocardium (Figure 3(a)).  Figure 3(b) illustrates that patients with cardiac inflammation showed a trend towards increased numbers of MSC (CD45^−^CD34^−^CD90^+^CD105^+^, 0.80 ± 0.67 versus 1.89 ± 1.03/100 mm^2^, *P* = 0.058) in their EMB compared to patients without inflammation and showed a strong correlation between MSC and cardiac inflammation (*r* = 0.64, *P* = 0.0001, not shown). Related to the number of cardiomyocytes, there were around 2 MSC/5000 cardiomyocytes. Time to diagnosis was identical in both subgroups (3.7 ± 2.3 versus 3.5 ± 3.2 month, *P* = 0.7). In agreement with the key role of SDF-1*α* in the retention of MSC, SDF-1*α* mRNA levels in EMB correlated with numbers of MSC in EMB (*r* = 0.64, *P* = 0.001, not show). Indeed, as shown in Figure 3(c), the numbers of MSC were significantly higher (*P* < 0.001) in the group of patients with SDF-1*α* mRNA expression above the median. By multivariate analysis, including LVEF, NT-proBNP, cardiac inflammation, and SDF-1*α*, SDF-1*α* was the strongest predictor for increased numbers of MSC in EMB (*P* < 0.001 for trend (ANOVA), *B* = 0.91, *P* < 0.005). These findings thus support a pivotal role of SDF-1*α* in the cardiac retention of MSC in the setting of CMi.

### 3.5. Mesenchymal Stromal Cells in Endomyocardial Biopsies and Transcardiac Gradients of Circulating Mesenchymal Stromal Cells

As shown in Figure 3(d), there was a significant inverse correlation between the number of MSC in EMB and transcardiac gradients of circulating MSC (*r* = −0.47, *P* < 0.05). Highest numbers of MSC in the EMB with the highest transcardiac gradients of circulating MSC are a first hint that MSC home to the myocardium. To further investigate whether the increased numbers of MSC in EMB were due to a potential replication of these cells in the heart, we analyzed Ki67 as nuclear marker for proliferation in MSC. As shown in the representative pictures in Figure 3(e), there was no costaining of Ki67 in MSC in EMB detectable, further supporting the constant migration of MSC. Due to the very rare number of Ki67+ cells, no further quantitative analysis could be performed.

### 3.6. SDF-1*α*- and MCP-1-Dependent Migration of Mesenchymal Stromal Cells* In Vitro*

**Figure 4:**
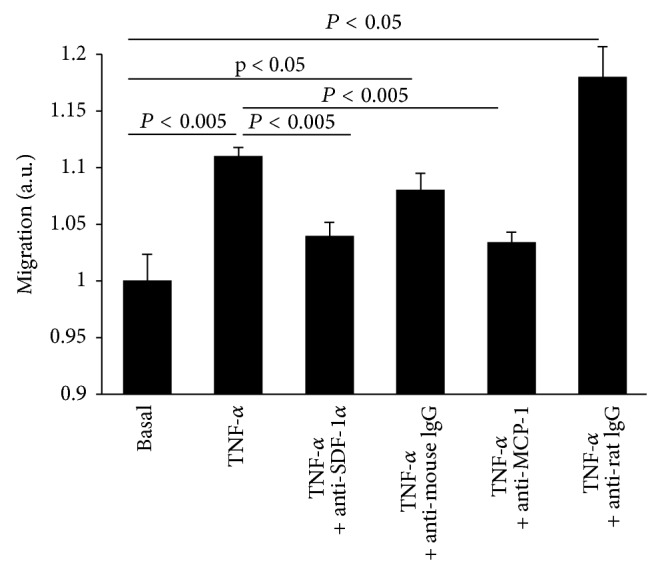
SDF-1*α*- and MCP-1-dependent migration of MSC* in vitro*. Migration of MSC (in arbitrary units) towards basal conditioned medium of HL-1 cardiomyocytes and that of HL-1 cardiomyocytes stimulated with TNF-*α*, in the presence or absence of a SDF-*α*-antibody, MCP-1-antibody, or IgG-antibody. Columns represent mean ± SEM with *n* = 4/group.

To assess the cardiac homing of circulating MSC towards inflamed hearts and to investigate which chemokines trigger the cardiac homing in patients with CMi, the migratory potential of MSC was determined towards conditioned medium of TNF-*α* stimulated HL-1 cardiomyocytes, to mimic the inflamed condition, in the presence or absence of a SDF-1*α*- or MCP-1 antibody. Medium of TNF-*α*-stimulated murine HL-1 cardiomyocytes increased the migration of MSC by 10% (*P* < 0.005) compared to basal conditioned medium of HL-1. This effect was impaired in the presence of an SDF-1*α*-antibody or in the presence of an MCP-1 antibody, but not in the presence of their respective IgG isotype controls (Figure 4).

## 4. Discussion

The salient findings of this study are that endogenous MSC migrate into the heart of patients with virus negative CMi depending on the grade of inflammation and that the number of retained cardiac MSC is related to the expression of SDF-1*α*. The increased number of MSC in the myocardium of patients with CMi is, at least in part, due to a constant recruitment of these cells.

Several studies in patients with heart failure have demonstrated the presence of humoral and cellular immunity activation, thus suggesting a possible relationship between dilated cardiomyopathy and inflammation [14]. In heart failure patients, inflammation is a strong predictor of negative outcome [2]. The nature of MSC with their immunomodulatory potential has moved these cells into the focus of novel therapeutic strategies for the treatment of heart failure. Recent data in experimental models of CMi [8, 13] underscore the potential of i.v. application of MSC to treat CMi. We and others have shown reduced cardiomyocytes injury in the setting of virus-induced acute myocarditis through paracrine and immunomodulatory effects of MSC [8, 13, 20], indicating beneficial effects of MSC in CMi. Most likely paracrine actions or modulation of regulatory immune cells account for the action of this admittedly small amount of MSC homed to the heart. This is in line with other findings from other progenitor cells present in small numbers in the heart exerting their effect on cardiac regeneration in the setting of ischaemia [21], advanced cardiomyopathies [20], and inflammation [22]. Given the significance of the cardiosplenic axis in the pathogenesis of CMi [13, 23] and heart failure [24], an important contribution of targeting of MSC to the spleen and subsequent immunomodulatory effects should be considered in the final cardioprotective effects of MSC after i.v. application.

At present, no clinical studies in patients with CMi have yet been performed involving i.v. administration of MSC and a direct proof that these multipotent cells play a physiological role in human CMi is lacking.

We therefore investigated whether endogenous circulating MCS migrate and home into the myocardium in CMi patients. In our study, there was a significant transcardiac reduction of circulating MSC in patients with cardiac inflammation. However, cardiac inflammation per se does not determinate numbers of cardiac MSC. The association of reduction of circulating MSC with increase of cardiac MSC is a hint towards homing of MSC to the site of injury in CMi. Here, we are in line with other studies [20] demonstrating that MSC selectively home to sites of injury, irrespective of the type of tissue.

Among different mechanisms leading to a continuous recruitment of MSC into the heart of patients with CMi, SDF-1*α* [25, 26] is known to be crucial for local retention of stem cells. Our findings are consistent with those results: the homing factor SDF-1*α* had the strongest association with numbers cardiac of MSC, as marker for cardiac retention, in our patients with CMi, whereas inflammation was the strongest determinant for continuous declined numbers of circulating MSC after transcardiac passage as indicator for migration. This could also be confirmed* in vitro* where SDF-1*α* was not shown as main contributor to migration. Furthermore, we did not see any proliferation of cardiac MSC, which supports our hypothesis that MSC are rather constantly recruited.

In brief, this study underscores the importance of the SDF-1*α*/CXCR4 axis in the cardiac homing of MSC by CMi. This finding as well as the observation that CXCR4 expression is decreased upon cell culture expansion [27] stresses the relevance to induce CXCR4 expression in MSC before injection. Due to the clinical nature of this study, further conclusions as to the major sources and orchestration of chemokines involved in MSC homing and retention and further mechanistic insights are precluded.

Furthermore, it has to be acknowledged that despite the well-documented cardioprotective effects of MSC in experimental models of CMi [8, 13], further studies will have to address the fate and function, repair of CMi or contribution to the pathogenesis of CMi, of endogenous cardiac MSC in humans.

## 5. Conclusion

We provide evidence that endogenous circulating MSC are constantly recruited into the myocardium of patients with CMi. It will be the aim of future studies to analyse the therapeutic potential of MSC in this disease.

## Figures and Tables

**Table 1:** Patients' baseline characteristics.

Parameter	Total population	No cardiac inflammation	Cardiac inflammation	Significance
*n*	29	6	23	
Age (years)	48 ± 16	48 ± 15	51 ± 14	n.s.
Male gender	16 (55%)	4 (67%)	12 (52%)	n.s.
NYHA class	4/14/8/2	1/4/1/0	3/10/7/2	n.s.
(I/II/III/IV), in %	(14/48/27/7)	(17/67/17/0)	(13/44/30/9)	
Duration of symptoms (month)	3.6 ± 2.4	3.5 ± 3.2	3.7 ± 2.3	n.s.
Cardiac risk factors				
Smoking	8 (28%)	3 (50%)	4 (17%)	n.s.
Diabetes mellitus	1 (3%)	0	1 (4%)	n.s.
Hypercholesterinaemia	11 (38%)	1 (17%)	10 (44%)	n.s
Arterial hypertension	16 (55%)	4 (67%)	12 (52%)	n.s.
Medication				
ACE-inhibitors/AT1-blocker	16 (55%)	4 (67%)	10 (44%)	n.s.
*β*-blocker	19 (66%)	3 (50%)	13 (57%)	n.s.
Statins	6 (21%)	1 (17%)	4 (17%)	n.s.
Haemodynamic and echocardiographic measurements				
LVEF (%)	52 ± 16	58 ± 22	49 ± 15	0.16
LVEDD (mm)	55.3 ± 8.1	51.6 ± 6.13	59.78 ± 11	0.04
Laboratory parameters				
Leukocyte count (/nl)	7.3 ± 2.3	6.4 ± 1.6	7.3 ± 1.9	n.s.
Hs-CRP (mg/dl)	1.9 ± 5.2	1.2 ± 1.8	2.1 ± 6.1	n.s.
NT-proBNP (pg/ml)	949 ± 1901	890 ± 949	1089 ± 2097	n.s.
Endomyocardial biopsy				
CD3 (/mm^2^)	18.8 ± 55.6	4 ± 2	23 ± 61	0.04
LFA-1 (/mm^2^)	27.5 ± 56.6	7 ± 2	33 ± 63	n.a.
HLA (AF%)	8.9 ± 5.0	7 ± 2	9 ± 6	n.s.

NYHA: dyspnoea according to the New York Heart Association; LVEF: left ventricular ejection fraction; LVEDD: left ventricular end diastolic diameter; hs-CRP: high sensitivity C-reactive protein; CD3: T-lymphocytes; LFA: lymphocyte function-associated antigen-1, CD11a; HLA: human leukocyte antigen; AF: area fraction. Significant *P* values are given for subgroup analyses.

## List of Abbreviations

CVB3: Coxsackievirus B3
CMi: Inflammatory cardiomyopathy
EMB: Endomyocardial biopsy
IL: Interleukin
i.v.: Intravenous
LVEF: Left ventricular ejection fraction
MSC: Mesenchymal stromal cell
MNC: Mononuclear cell
MCP-1: Monocyte chemoattractant protein-1
NT-proBNP: N-Terminal pro-brain natriuretic peptide
PBMCs: Peripheral blood mononuclear cells
PCR: Polymerase chain reaction
SDF-1*α*: Stromal derived factor-1*α*
TCG: Transcardiac gradient.
